# Novel mutations in DNA2 associated with myopathy and mtDNA instability

**DOI:** 10.1002/acn3.50888

**Published:** 2019-09-02

**Authors:** Dario Ronchi, Changwei Liu, Leonardo Caporali, Daniela Piga, Hongzhi Li, Francesca Tagliavini, Maria Lucia Valentino, Maria Teresa Ferrò, Paola Bini, Li Zheng, Valerio Carelli, Binghui Shen, Giacomo Pietro Comi

**Affiliations:** ^1^ Neurology Unit Fondazione IRCCS Ca' Granda Ospedale Maggiore Policlinico Milan Italy; ^2^ Dino Ferrari Center Department of Pathophysiology and Transplantation University of Milan Milan Italy; ^3^ Department of Cancer Genetics and Epigenetics Beckman Research Institute City of Hope Duarte California; ^4^ Istituto delle Scienze Neurologiche di Bologna UOC Clinica Neurologica Bologna Italy; ^5^ Dipartimento di Scienze Biomediche e Neuromotorie (DIBINEM) Università di Bologna Bologna Italy; ^6^ Neurology Unit Ospedale Maggiore Crema Italy; ^7^ IRCCS "C. Mondino" Foundation National Neurological Institute Pavia Italy; ^8^ Department of Neuroscience, Neuromuscular and Rare Diseases Unit Fondazione IRCCS Ca' Granda Ospedale Maggiore Policlinico Milan 20122 Italy

## Abstract

The maintenance of mitochondrial DNA (mtDNA) relies on proteins encoded by nuclear genes. Mutations in their coding sequences result in heterogenous clinical presentations featuring mtDNA instability in affected tissues. DNA2 is a multi‐catalytic protein involved in the removal of single strand DNA during mtDNA replication or Long Patch Base Excision Repair pathway. We have previously described *DNA2* mutations in adult patients affected with familial and sporadic forms of mitochondrial myopathy. Here we describe four novel probands presenting with limb weakness associated with novel *DNA2* molecular defects. Biochemical assays were established to investigate the functional effects of these variants.

## Introduction

Several mechanisms occur in the nuclear and mitochondrial (mtDNA) genome to preserve DNA integrity. Mutations in nuclear genes encoding enzymes devoted to mtDNA homeostasis result in heterogenous clinical presentations collectively termed “mtDNA maintenance disorders”.^1^ These enzymes often feature multiple catalytic activities engaged during mtDNA repair, replication or transcription.^2^

An example is DNA2, a multi‐catalytical (helicase/nuclease) protein found in mammalian mitochondria, where it participates in the removal of damaged bases in the Base Excision Repair (BER) pathway and the removal of RNA primers during mtDNA replication.^3^ We have previously identified *DNA2* missense mutations in adult patients presenting with progressive myopathy and muscle mtDNA deletions (MIM 615156).^4^ Since then, *DNA2* mutations have been detected in Seckel syndrome (MIM 615807)^5^ and congenital myopathy.^6^, ^7^

Here we further expand the number of cases harboring *DNA2* defects.

## Subjects and Methods

### Subjects

The study was approved by the local ethics committees and was performed in accordance with the Declaration of Helsinki. Informed consent was obtained from all participants. Clinical, instrumental and molecular findings are summarized in Table 1. Family pedigrees are shown in Figure S1.

**Table 1:** Clinical, instrumental and molecular findings of the patients described in the study.

Patient	Gender	Age (years)	Age at Onset (years)	Clinical Features	EMG	Muscle Biopsy	DNA2 mutation	mtDNA
P1	F	76	34	Ptosis, myalgia, diabetes, cataract	N	COX‐ (0.17%) RRF (0.03%)	c.1919C > T, p.Ser640Leu	mtDNA dels (SB, long range PCR)
P2	M	56	30	Limb‐girdle weakness, hypotonia, peripheral neuropathy, cataract.	M	COX‐ (2.3%) RRF (2.9%)	c.2867G > A, p.Arg956His	mtDNA dels (SB, long range PCR)
P3	F	70	65	Ptosis	N	COX‐ (0.2%)	c.1655C > T, p.Ser552Leu	mtDNA dels (long range PCR)
P4	F	64	57	Ptosis, multiple sclerosis	n.a.	COX‐ (0.4%)	c.662C > G, p.Ala221Gly	mtDNA dels (long range PCR)

EMG, electromyography; N, normal; M, myopathic; n.a, not assessed; COX‐, Cytochrome c Oxidase negative fibers; RRF, Ragged Red fibers; mtDNA dels, mitochondrial DNA multiple deletions; SB, Southern Blot.

Patient 1 came to our attention at 76 years of age. She displayed bilateral ptosis since the age of 34. At 54 years she complained of back pain, muscle cramps and walking difficulties. She underwent surgery for endometrial cancer. Additional symptoms included arterial hypertension, essential tremor, cataract and diabetes. Her mother was asymptomatic. Her father also had bilateral ptosis and tremor. She died from lung cancer at 83 years of age. Family history of paternal lineage was positive for lung and gastrointestinal neoplasia. The proband’s younger sister (now 68 years old) also complains of back pain and walking difficulties. The proband’s daughter is asymptomatic while her son suffers of back pain. Muscle biopsy (biceps), performed at 70 years of age, showed the presence of scattered (0.17%) COX‐negative/SDH‐positive fibers and a single Ragged Red fiber (0.03%).

Patient 2 is a 56‐year‐old man with a 6‐year history of progressive limb‐girdle muscle weakness, lower limb hypotonia and exercise intolerance. He walks with a walker. Additional symptoms include peripheral neuropathy and cataract. His family history is unremarkable for neuromuscular disorders. His neurological conditions remained relatively stable in the last 10 years.

His mother died at the age of 52 year from liver cancer. COX‐negative (2.3%) and Ragged Red fibers (2.9%) were detected at muscle biopsy (quadriceps), performed at the age of 56 years.

Patient 3 is a 70‐year‐old woman with a 5‐year history of mild bilateral ptosis, without ophthalmoplegia, revealed by ophthalmological examination and probably present since a few years before. Her family history was negative for ptosis. She underwent electrophysiological and laboratory analysis that excluded myasthenia. Her neurological examination was unremarkable, except for bilateral ptosis. Laboratory exams showed a mild hyper‐CKemia (232 U/L, n.v. <145 U/L). Muscle biopsy (biceps), performed at 69 years of age, revealed a few COX‐negative fibers (0.2%).

Patient 4 is a 64‐year‐old woman with a history of relapsing/remitting multiple sclerosis that started at 50 years and was treated with interferon and corticosteroids. At 57 years she developed ptosis in the right eye, followed by ptosis in the left eye some years later, slowly progressive, without diplopia. Electrophysiological and laboratory analysis excluded myasthenia. Her neurological examination showed a bilateral ptosis, without ophthalmoplegia and spastic paraparesis. Family history was negative for neurological disorders. Muscle biopsy (biceps), performed at 64 years of age, showed scattered COX‐negative fibers (0.4%).

### Methods

Genes involved in mtDNA maintenance disorders were ruled out using direct sequencing (Patients 1 and 2) or gene‐panel sequencing (Patients 3 and 4) starting from blood‐derived DNA. Coding exons and intronic boundaries of human DNA2 (NM_001080449.2) were analyzed as described.^4^ Enzymatic studies performed on purified recombinant DNA2 proteins were performed as previously described.^3^, ^4^ Additional details on experimental procedures are included in the Data S1 section.

### Results

**Figure 1:**
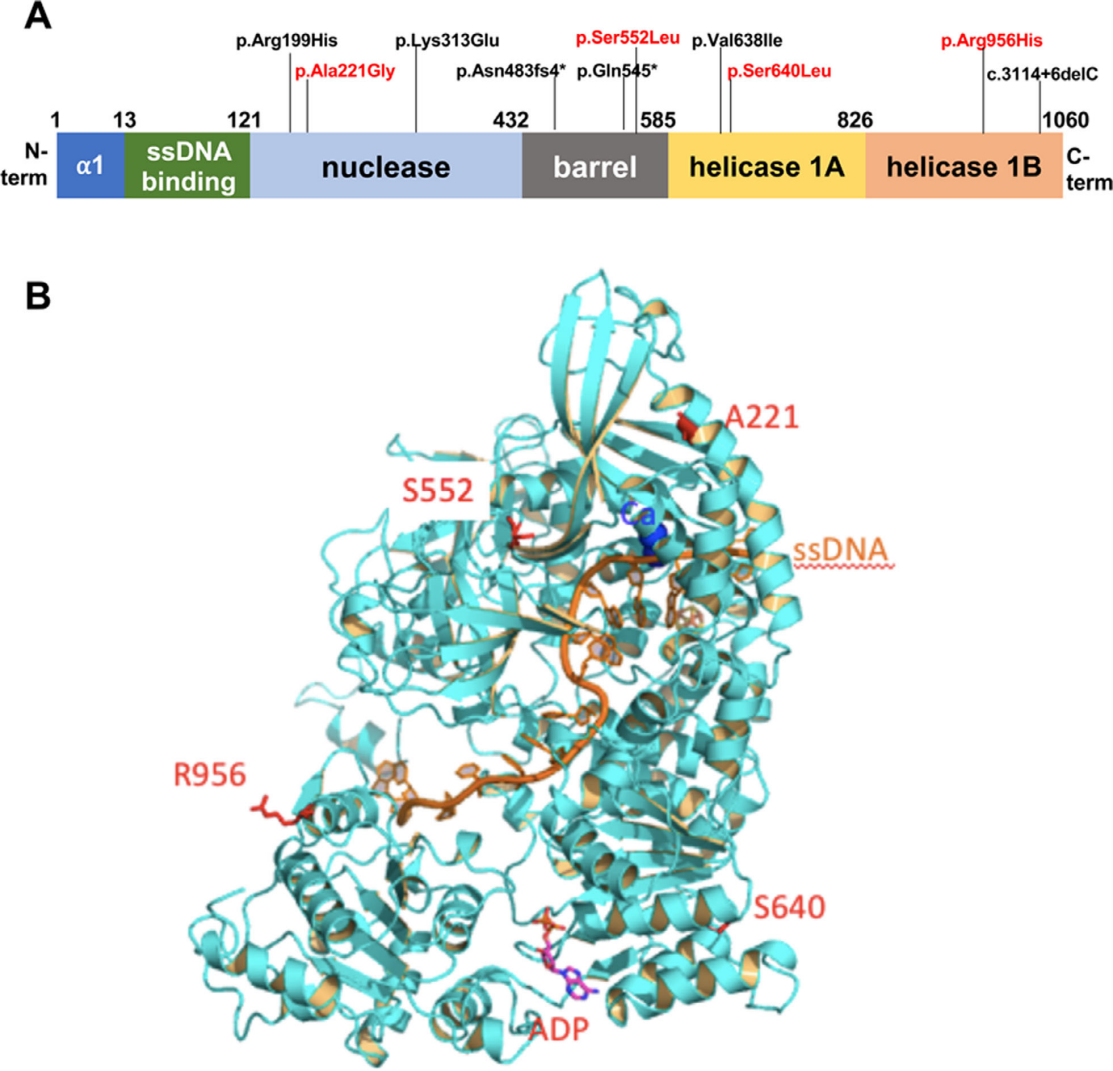
Novel DNA2 variants identified in this study. (A) A scheme of human DNA2 including the location of the mutations so far identified in the coding sequence (red color indicates the novel variants presented here). The diagram shows the functional domains conserved in this enzyme. (B) Homology model of DNA2 with adenosine diphosphate (ADP) (pink) binding in the catalytic center of the ATPase domain (blue) interacts with ssDNA (orange). The positions of four residues, Ala221, Ser 640, Ser552 and Arg956, are illustrated as red sticks.

All probands displayed multiple mtDNA deletions in muscle samples detected by Southern blot or long range PCR analysis (Fig. S2). *DNA2* sequencing revealed the presence of the following heterozygous mutations (Fig. 1A), resulting in the amino acid substitutions: c.1919C > T (p.Ser640Leu, Patient 1), c.2867G > A (p.Arg956His, Patient 2), c.1655C > T (p.Ser552Leu, Patient 3) and c.662C > G (p.Ala221Gly, Patient 4). Two variants displayed very low frequency in gnomAD database (https://gnomad.broadinstitute.org c.2867G > A: 4.2E‐06 and c.662G > C: 1.37E‐05).

Segregation analysis was positive in the available affected relatives of Patient 1, whereas no mutation was detected in the asymptomatic daughter.

**Figure 2:**
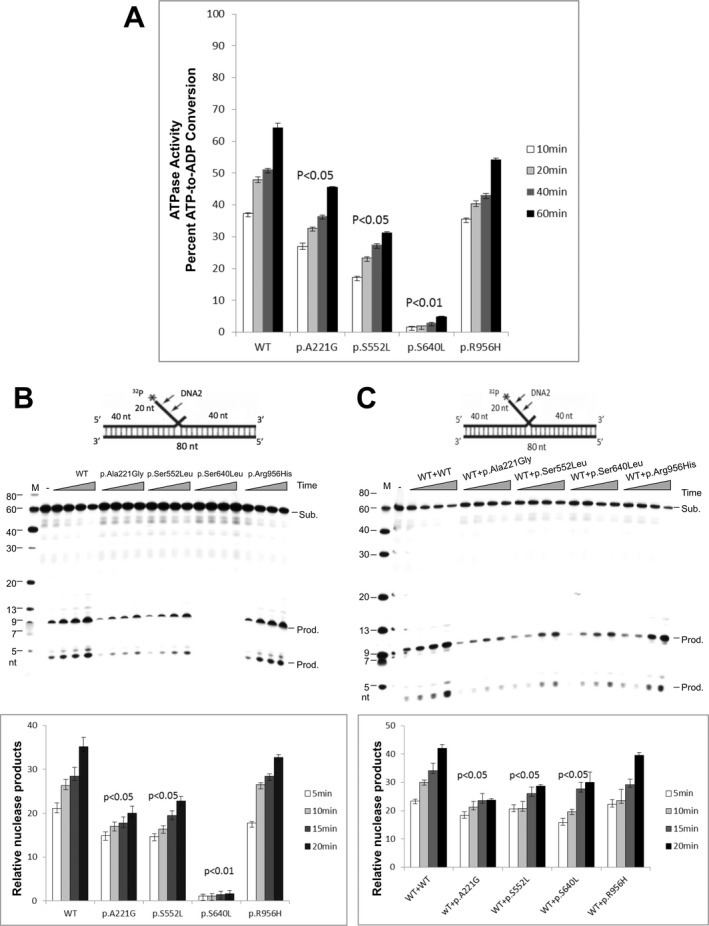
Biochemical studies (A) ATPase activity of WT and altered forms of DNA2. 100 ng WT and mutants of DNA2 were incubated with 1 mmol/L ATP and 1 *μ*g 22 bp ssDNA for 10, 20, 40 and 60 min, respectively. ATPase activity of WT or mutant DNA2 proteins was assayed using the adenosine diphosphate (ADP)‐Glo™ Max Assay kit (Promega). Values are means ± SEM of ATPase activities (percent of ATP‐to‐ADP conversion) of three independent assays. (B) Endonuclease activities of WT and altered forms of DNA2. Top panel is the substrate mimicking Flap Okazaki fragment. Arrows indicate cleavage sites. 0.5 ng of WT, p.Ala221Gly, p.Ser552Leu, p.Ser640Leu and p.Arg956His were respectively incubated with 0.5 pmol 5′‐end ^32^P‐labeled substrate. Reactions were performed at 37°C for 5, 10, 15 and 20 min. “Sub.” indicates substrate, “Prod.” indicates cleavage product. Bottom panel shows the quantification of DNA2 cleavage products. The relative nuclease activity was calculated by dividing the intensity of the product bands by the sum of the intensities of product and substrate bands in each reaction. Values are means ± SEM of nuclease activities (percent of cleavage products) of three independent assays. (C) Heterogeneity effects of the nuclease activity of altered forms of DNA2. 0.25 ng WT DNA2 protein was mixed with 0.25 ng p.Ala221Gly, p.Ser552Leu, p.Ser640Leu and p.Arg956His, respectively. The mixture was incubated with 0.5 pmol 5′‐end ^32^P‐labeled substrate. Reactions were performed at 37°C for 5, 10, 15 and 20 min. Bottom panel shows the quantification of DNA2 cleavage products. Values are means ± SEM of three independent assays. In each panel, the *P*‐value was calculated using a Student’s *t* test.

The mutations affect residues well conserved in mammalian *DNA2* orthologues, with the exception of p.Arg956 (Fig. S3). The tridimensional modeling of DNA2 was used to predict the potential consequences of the identified variants on protein structure and activity (Fig. 1B). We also expressed and purified recombinant wild type and mutant DNA2 proteins to check the impact of the mutations on nuclease and ATPase activities (Fig. 2A and 2B).

The amino acid change Ser640Leu in helicase 1A domain results in the loss of the hydrogen bond with Arg781, hampering the binding of ssDNA. The mutation was found to abolish both nuclease and ATPase activities.

The Arg956His mutation falls within the helicase 2A domain and it is predicted to affect the ssDNA binding via allosteric effect on positively charged residues Arg944 and Lys968. Biochemical studies did not show a significant impact on DNA2 activities.

The substitution Ala221Gly affects the nuclease domain and may weaken core hydrophobic interaction for a helix bundle that contacts the nuclease catalytic center. Biochemical studies confirmed a modest, but significant, decrease of both endonuclease and ATPase activities compared to wild type protein.

The change Ser552Leu affects the barrel domain, resulting in the loss of the hydrogen bond with Asn550 likely leading to destabilization of the loop structure and weakening the ssDNA binding domain. Both nuclease and ATPase activities are affected by the presence of the variant.

To mimic the heterozygous nature of the variants, we also mixed the WT DNA2 with equal amounts of WT and mutant forms of the protein (Fig. 2C). Compared to reactions with WT/WT proteins, the product of reactions of WT/Ser640Leu, WT/Ala221Gly and WT/Ser552Leu were decreased supporting the hypothesis that WT DNA2 cannot fully compensate the detrimental effects of the mutations.

## Discussion

In this study we expanded the list of *DNA2* mutations linked with adult onset presentations. Clinical features of the novel probands mainly show muscle involvement (ptosis, muscle weakness). Peripheral neuropathy, diabetes and cataract were also observed. A heterogenous COX‐negative pattern was observed at muscle biopsy. COX‐negative fibers were especially rare in Patients 1, 3 and 4 in which muscle involvement was restricted to extraocular muscles. This finding can be attributed to secondary age‐related changes or, more likely, to the limited informativeness of the muscles sampled. Indeed, extraocular muscles tend to show a greater prevalence of COX‐negative and RRF fibers compared to limb muscles in adult subjects.^8^

Heterozygous truncating mutations have been recently associated with pediatric presentation featuring congenital muscle hypotonia, with ptosis^6^ or multiple joint fractures.^7^ A homozygous truncating mutation was proposed as the underlying cause of a form of dwarfism (Seckel syndrome) in a Saudi Arabian pedigree showing consanguinity.^5^ It is likely that severe mutations affecting in part (haploinsufficiency) or totally abrogating *DNA2* expression might show profound consequences on DNA2 activity, which in turn reflects on mtDNA content (severe mtDNA depletion), leading to congenital severe presentations.

As we previously observed,^4^ DNA2 missense mutations often produce a detrimental effect on multiple domains impairing both nuclease and ATPase activities. In this study only the DNA2 Arg956His mutation did not differ from wild type enzyme. This variant lies in the helicase domain 2A and it is predicted to alter the binding of single strand DNA, hampering DNA replication. Although helicase function was considered dispensable for the DNA end resection, recent studies suggest this domain might facilitate the traversal of DNA2 over the RNA primer associated with Okazaki fragments.^9^ We cannot exclude that the variant identified in Patient 2 might impact this mechanism, which still needs to be confirmed for mtDNA. In this regard, the study of the biochemical defects underlining mitochondrial disorders featuring mtDNA instability has increased our knowledge of the proteins involved in mtDNA replication and repair such as DNA2 and its partners MGME1^10^ and RNaseH1.^11^

Abnormal regulation of DNA2 activity might also result in nuclear DNA stress, impairment of its repair mechanism and genomic instability as supported by experiments in reconstituted systems and in human cells.^12^, ^13^ Indeed, downregulation^14^ or overexpression^15^ of DNA2 activity have been observed in human tumors. It is tempting to speculate that even germline inherited *DNA2* mutations might predispose to cancer, as suggested by the high incidence of different forms of neoplasia in Patient 1’s paternal relatives.

Mutations in *DNA2* are a rare cause of mitochondrial disease, with independent mutated subjects now representing the 2.7% of our cohorts of adult patients with mtDNA maintenance disorders (5.2% of undiagnosed cases). The prevalence of limb‐girdle weakness prompts to include *DNA2* analysis in diagnostic gene panels for neuromuscular patients, even in the absence of additional clues directing towards mitochondrial dysfunction.

## Conflict of Interest

The authors declare that they have no conflict of interest.

## Supporting information


**Data S1** **.** Supplementary materials and methods.
**Figure S1** **.** Pedigree of the probands described in the paper.
**Figure S2** **.** Multiple mtDNA deletions in patients’ muscle.
**Figure S3** **.** Alignment for the DNA2 protein sequences.
