# Supplementary material for: Novel mutations in DNA2 associated with myopathy and mtDNA instability
Source: Ann Clin Transl Neurol. 2019 Sep 2;6(9):1893–9. doi: 10.1002/acn3.50888 (PMC6764641; doi:10.1002/acn3.50888)
Supplement: Supplementary file 1 — Data S1 . Supplementary materials and methods. Figure S1 . Pedigree of the probands described in the paper. Figure S2 . Multiple mtDNA deletions in patients’ muscle. Figure S3 . Alignment for the DNA2 protein sequences. [file ACN3-6-1893-s001.docx]

Novel Mutations in DNA2 associated with myopathy and mtDNA instability

Supplementary Material

**Supplementary Methods** Supplementary materials and methods. Page 2

**Supplementary Figure 1** Pedigree of the probands described in the paper. Page 3

**Supplementary Figure 2** Multiple mtDNA deletions in patients’ muscle. Page 4

**Supplementary Figure 3** Alignment for the DNA2 protein sequences. Page 5

**Supplementary Methods**

**Molecular studies**

Sanger sequencing was used to investigate coding regions of genes associated with mtDNA maintenance disorders (Ahmed at al. Int J Mol Sci. 2015 Aug 5;16(8):18054-76) in Patients 1 and 2.

Patients 3 and 4 were investigated by a panel including 25 genes associated with mtDNA instability (list available upon request). The library was generated using a 250 bp amplicon-based approach (TruSeq Custom Amplicon, Illumina) and sequenced on MiSeq instrument (Illumina). Reads were aligned to the human genome (assembly hg19) and the identified variants were annotated (ANNOVAR) and filtered, focusing on rare variants (≤0.5% in public databases), causing changes potentially damaging for the protein function (CADD, DANN).

**Expression and purification of wild-type (WT) and mutant recombinant DNA2 proteins**

The DNA fragment encoding WT or mutant human DNA2 was subcloned into the p3xflag CMV7.1 plasmid (Sigma). The plasmid for DNA2 expression was transfected into 293T cells. After 48 hours, the cells were lysed and WT or mutant 3xFlag-DNA2 proteins were purified using the anti-Flag M2 beads (SIGMA) following the manufacturer’s instructions. Briefly, cell lysates were incubated with the beads for at 16 h at 4 °C. After extensively washing with a buffer containing 50mM Tris-Cl (pH 7.5) and 500mM NaCl, the bound 3xFlag-DNA2 proteins were eluted with 3xFlag peptide. The free 3xFlag was removed and the protein was concentrated using the centrifugal filter device. The purity of the recombinant proteins was evaluated by SDS-PAGE electrophoresis.

**ATPase activity assay**

ATPase activity assay was measured by the ADP-Glo™ Max Assay kit (Promega) following manufacturer instructions. In brief, 100 ng WT and mutants DNA2 proteins were incubated with 1 mM ATP and 1 ug 22bp ssDNA for a specific time period, respectively. After DNA2-driven ATPase reaction, the ADP-Glo™ Reagent was added to the mixture to stop the reaction and to deplete the remaining ATP as well. The detection solution was then added to detect the ADP level based on a luciferase/luciferin reaction. The ADP level was determined by comparing light generated in the DNA2 reaction to the light generated in the ADP standard solution. The percentage of ATP that was converted to ADP was calculated by dividing the ADP level to the original ATP level. Three independent assays were conducted and the DNA2 activity. The activity was expressed as means ± SEM of the percent of ATP-to-ADP conversion.

**Nuclease activity assay**

DNA2 nuclease activity was assayed as previously described (Zheng L et al. Mol Cell. 2008 Nov 7;32(3):325-36). Briefly, to make the flap DNA substrate, the flap strand oligo was labelled with ^32^P at the 5’ end. After purification by Ethanol precipitation, it was annealed to the upstream strand oligo and template strand oligo at 1:1.5:2 ratio. To compare the nuclease activity of WT or mutant DNA2 proteins, the WT or mutant enzyme (0.5 ng each reaction) was incubated with DNA substrates (0.5 pmol each reaction) in the reaction buffer containing 50mM Hepes-KOH (pH 7.5), 5 mM MgCl_2_, 2 mM DTT, and 0.1 mg/ml BSA at 37 °C for a specific time period. The DNA substrates and products were resolved with a 15% denaturing PAGE and visualized by radio autography. The band intensities of substrate and product in each reaction were quantified with the ImageJ software. Three independent assays were conducted and the DNA2 activity. The activity was expressed as means ± SEM of the percentage of cleavage products (relative nuclease products).


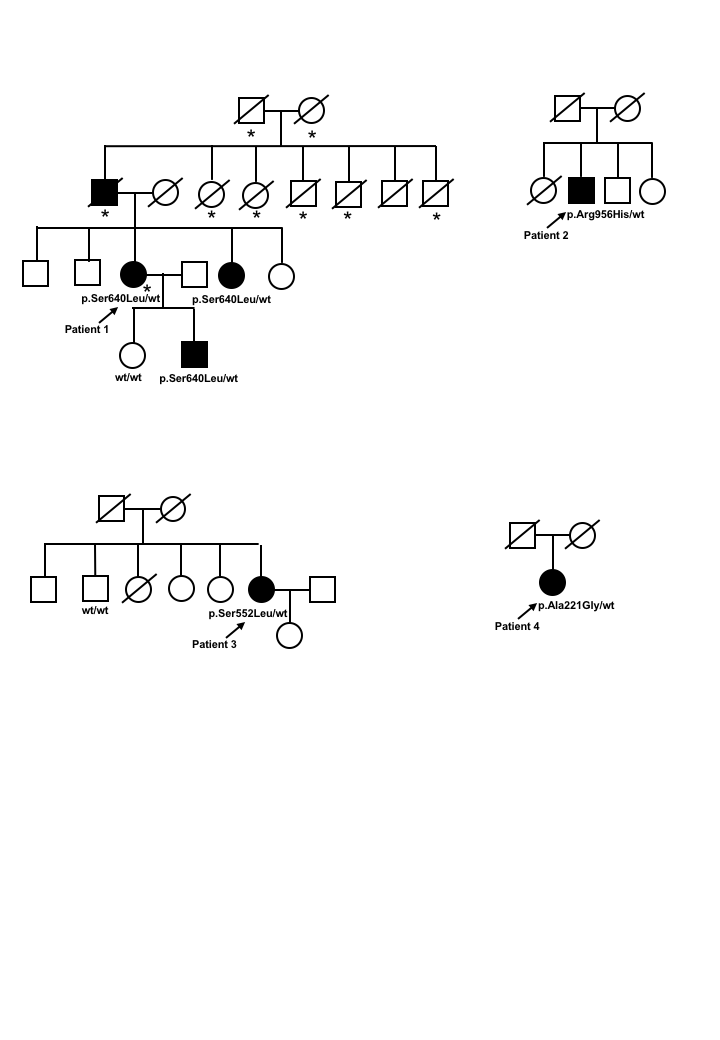
**Supplementary Figure 1.** Pedigree of the probands described in the paper. Arrows indicate index cases. Black symbols indicate affected individuals (isolated myopathy or with additional symptoms). Asterisks indicate a clinical history for neoplasia. Available genotypes are indicated below each subject.


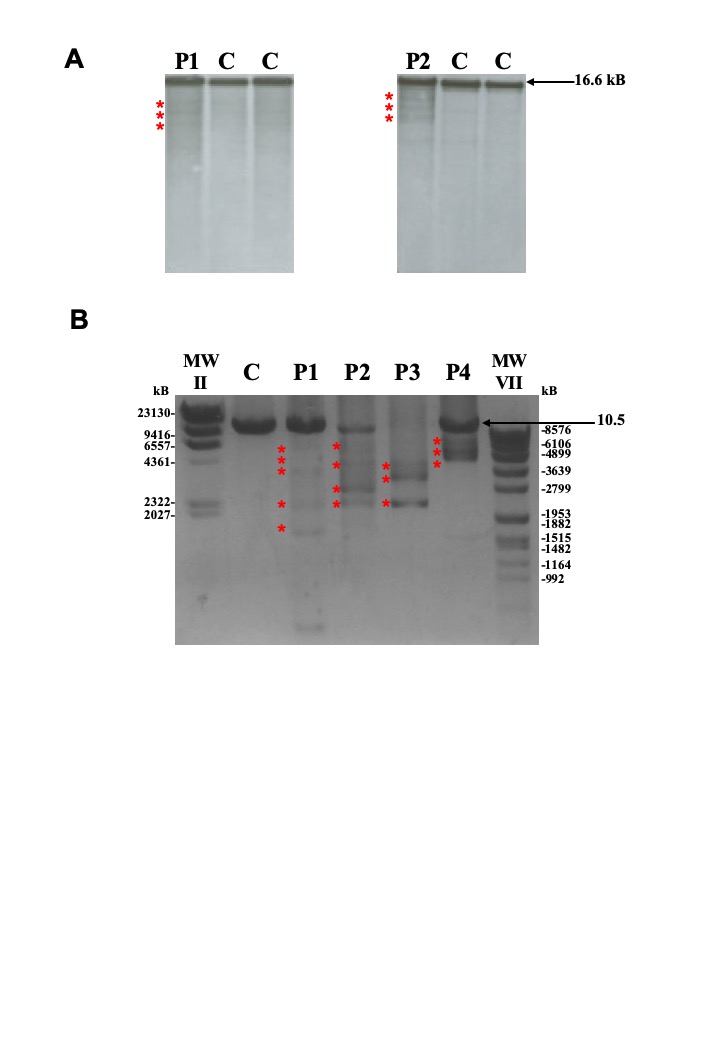


**Supplementary Figure 2.** Multiple mtDNA deletions in patients’ muscle. (**A**) Southern blot analysis of mitochondrial DNA obtained from patients’ muscle biopsies (P1 and P2) and age matched controls (C). Red asterisks indicate multiple bands corresponding to partially deleted mitochondrial genomes. Black arrows indicate the expected size of normal linearized mtDNA. (**B**) Long range PCR analysis of mitochondrial DNA obtained from patients’ muscle biopsies (P1-P4) and age matched control (C). Red asterisks indicate multiple bands corresponding to multiple mtDNA deletions. Black arrow indicates the expected size of wild-type PCR amplicon (10.5kB: FOR5635-RC16135). The sizes of the bands of the ladders (DNA Molecular Weight Marker II and VII, Roche) are indicated.


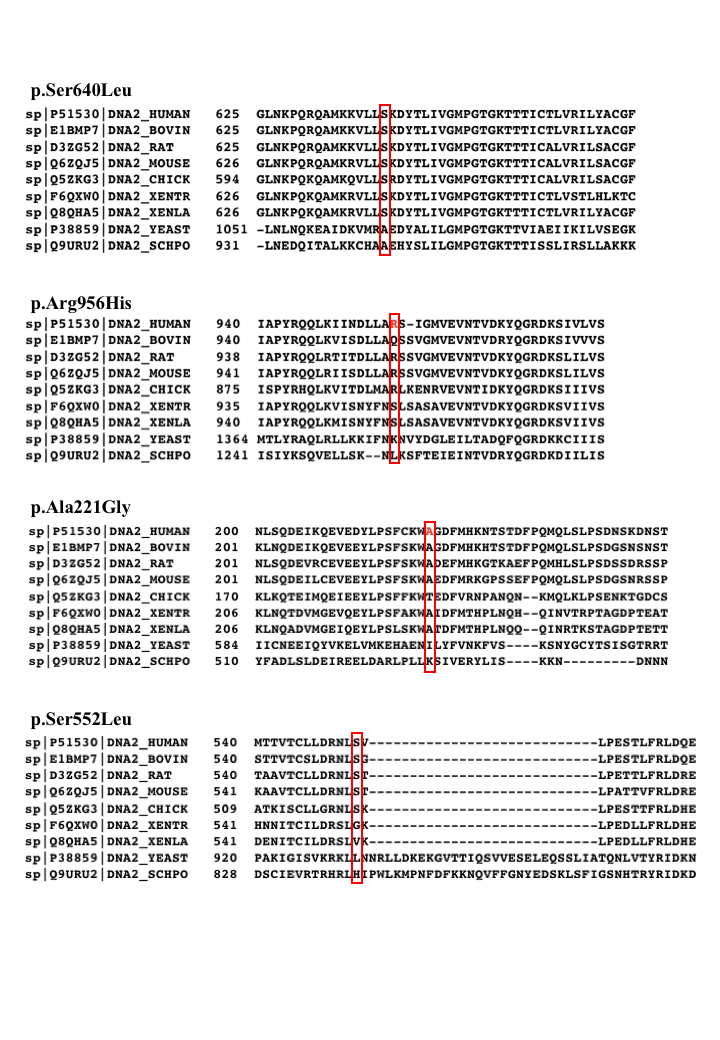


**Supplementary Figure 3.** Multiple-sequence alignment of amino acids for the DNA2 human and orthologous protein sequences with the use of CLC Bio Main Workbench v.5.1.
